# Sodium channel Nav1.7 in vascular myocytes, endothelium, and innervating axons in human skin

**DOI:** 10.1186/s12990-015-0024-3

**Published:** 2015-05-09

**Authors:** Frank L Rice, Phillip J Albrecht, James P Wymer, Joel A Black, Ingemar SJ Merkies, Catharina G Faber, Stephen G Waxman

**Affiliations:** Integrated Tissue Dynamics, LLC, Rensselaer, NY 12144 USA; Department of Neurology, Albany Medical College, Albany, NY 12209 USA; Center for Neuroscience & Regeneration Research, Yale University School of Medicine, West Haven, CT 06516 USA; Rehabilitation Research Center, VA Connecticut Healthcare System, West Haven, CT 06516 USA; Department of Neurology, Spaarne Hospital, Hoofddorp, the Netherlands; Department of Neurology, Maastricht University Medical Center, Maastricht, the Netherlands

**Keywords:** Arteriole-venule shunt, Cutaneous arterioles, Dermis, Smooth muscle cells, Sodium channels, Vascular myocytes

## Abstract

**Background:**

The skin is a morphologically complex organ that serves multiple complementary functions, including an important role in thermoregulation, which is mediated by a rich vasculature that is innervated by sympathetic and sensory endings. Two autosomal dominant disorders characterized by episodes of severe pain, inherited erythromelalgia (IEM) and paroxysmal extreme pain disorder (PEPD) have been directly linked to mutations that enhance the function of sodium channel Nav1.7. Pain attacks are accompanied by reddening of the skin in both disorders. Nav1.7 is known to be expressed at relatively high levels within both dorsal root ganglion (DRG) and sympathetic ganglion neurons, and mutations that enhance the activity of Nav1.7 have been shown to have profound effects on the excitability of both cell-types, suggesting that dysfunction of sympathetic and/or sensory fibers, which release vasoactive peptides at skin vasculature, may contribute to skin reddening in IEM and PEPD.

**Results:**

In the present study, we demonstrate that smooth muscle cells of cutaneous arterioles and arteriole-venule shunts (AVS) in the skin express sodium channel Nav1.7. Moreover, Nav1.7 is expressed by endothelial cells lining the arterioles and AVS and by sensory and sympathetic fibers innervating these vascular elements.

**Conclusions:**

These observations suggest that the activity of mutant Nav1.7 channels in smooth muscle cells of skin vasculature and innervating sensory and sympathetic fibers contribute to the skin reddening and/or pain in IEM and PEPD.

## Introduction

The skin is a morphologically complex organ that serves multiple complementary functions [1]. While fulfilling a protective role, the skin is an exquisite tactile sense organ designed to detect a wide variety of mechanical, thermal, chemical, and noxious stimuli over a wide range of intensities. In humans, the skin, particularly of the hands and feet, also plays an important role in thermoregulation [2-5]. These varied functions are subserved through a mix of discrete structures including the epidermis, dermal papillae, and a rich vasculature that are innervated by a variety of sympathetic and sensory nerve endings. While providing a high degree of versatility, the complexity of the skin and its innervation contributes to susceptibility to sensory neuropathies and sudomotor disorders associated with intractable chronic pain including diabetic neuropathy, postherpetic neuralgia, and chemotherapy-induced peripheral neuropathy [6-13].

Two autosomal dominant disorders characterized by episodes of severe pain, inherited erythromelalgia (IEM) [14,15] and paroxysmal extreme pain disorder (PEPD) [16,17], have been directly linked to mutations that enhance the function of sodium channel Nav1.7. Gain-of-function mutations of Nav1.7 have also been identified in some patients with painful small-fiber neuropathy [18]. In IEM and PEPD [15,17,19] and in some patients with small fiber neuropathy [20], episodes of pain are accompanied by reddening of the skin. Neurogenic [14,15] and vasogenic [21,22] mechanisms, and an abnormality of intracutaneous arteriole-venule shunting [23], have been suggested to contribute to the pathophysiology of EM. Consistent with a neurogenic mechanism, Nav1.7 is known to be expressed at relatively high levels within both dorsal root ganglion (DRG) and sympathetic ganglion neurons [24,25].

Mutations that enhance the activity of Nav1.7 have been shown to have profound effects on the excitability of both DRG neurons and sympathetic ganglion neurons [25,26], suggesting that dysfunction of sympathetic ganglion neurons may contribute to skin reddening in IEM, PEPD, and small fiber neuropathy [25]. However, while the microanatomy of normal and pathological human skin have been extensively studied [2,27], to date, the expression of Nav1.7 within intracutaneous vasculature and in the innervation of intracutaneous vasculature has not been studied. In this study, we demonstrate the presence of Nav1.7 within vascular myocytes of human intracutaneous arterioles and arteriole-venule shunts (AVS) of normal human glabrous skin, and skin from 10 cm above the lateral malleolus, a standard site for diagnostic and experimental skin biopsy [28]. We also demonstrate the presence of Nav1.7 within endothelium and in both the sensory and sympathetic innervation that converge and terminate on the intracutaneous vasculature.

## Results

### Nav1.7 expression in cutaneous arterioles and AVS

#### Vascular myocytes

**Figure 1:**
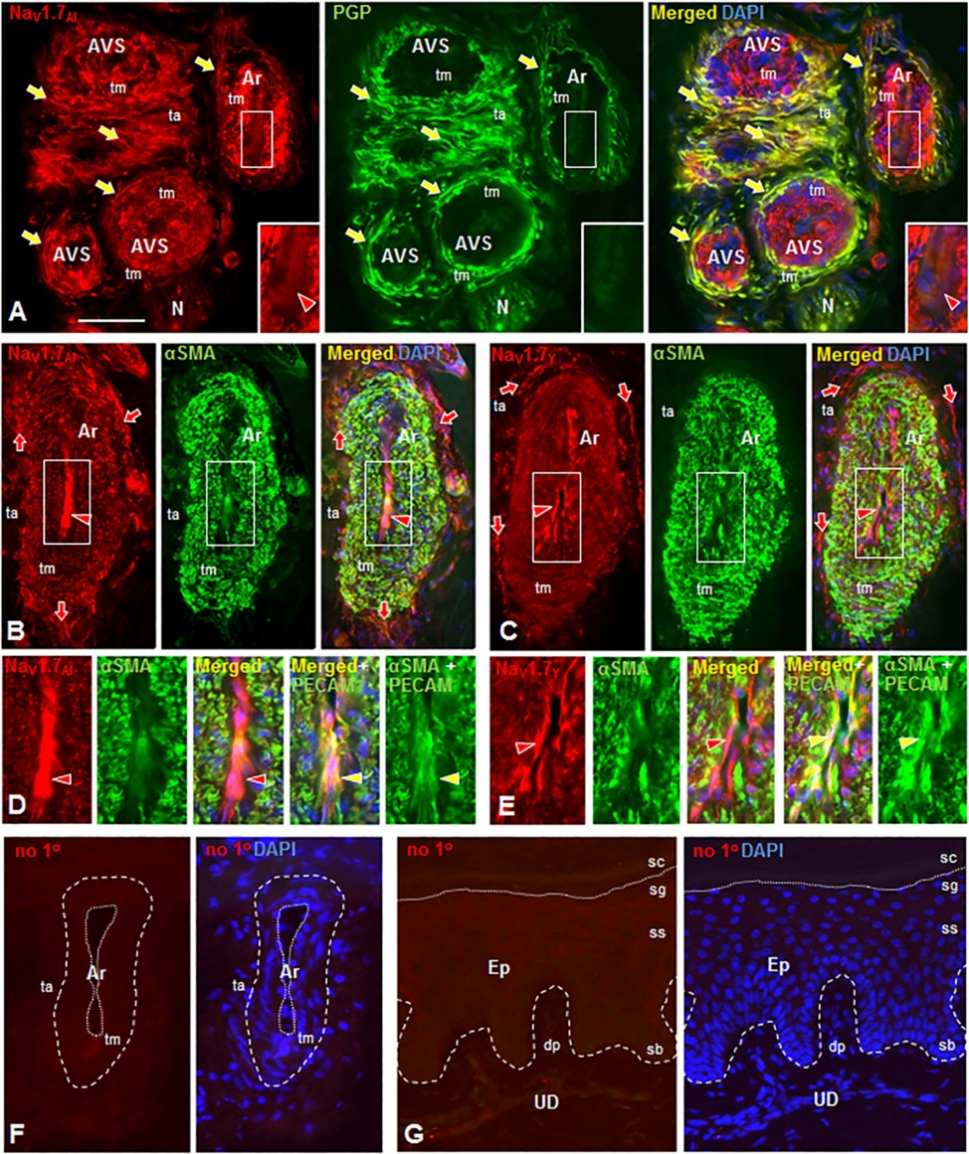
Nav1.7 immunolabeling (IL) of arterioles (Ar), arteriole-venule shunts (AVS) and associated innervation in normal human plantar glabrous skin with Alomone **(A,** **B)** or Yale **(C)** Nav1.7 antibodies (red). Co-labeling of innervation (arrows) as marked with anti-PGP 9.5 (PGP, green,** A**) or smooth muscle cells in tunica media (tm) as marked with anti α-smooth muscle actin antibody (αSMA, green, **B**,**C**). Nuclei are DAPI-labeled (blue). Left images (each panel) show only red fluorescence, middle images green; right images show triple-label combinations. Large white rectangles are 2X-enlargements of small rectangles. **A-C**. Nav1.7-IL is expressed on endothelial cells of tunica intima (red arrowheads) and tm smooth muscle cells as confirmed by double-labeling with anti-αSMA **(B,** **C)**. Nav1.7-IL is expressed on virtually all vascular innervation (arrows) in tunica adventitia (ta) as confirmed by anti-PGP 9.5 double-labeling (**A**, yellow arrows). N=nerve. **D-E**. Nav1.7-IL on arteriole endothelial cells shown as 2X-enlargements of areas indicated by white rectangles in **B**,**C**. First images (each panel) show Nav1.7-IL on smooth muscle cells in tm and endothelial cells (red arrowheads). The second images show α-SMA co-labeling of only the smooth muscle cells of tm (green). The third images show merge of first and second images with DAPI (blue). Sections re-labeled with anti-PECAM (green) to show co-labeling with Nav1.7 on endothelial cells (yellow arrowheads, fourth and fifth images). **F-G**. Background Cy3 fluorescence is limited with no primary antibody in arteriole deep in dermis **(F)**, epidermis (Ep) and upper-dermis (UD) **(G)**. In **F**, broken line shows tm perimeter with dotted line around arteriole lumen. In **G**, broken line indicates basement membrane of epidermis and dotted line indicates boundary of dead and live superficial keratinocyte layers (stratum corneum, sc and stratum granulosum, sg, respectively). Stratum spinosum, ss; stratum basalis, sb; dermal papilla (dp). Scale bars=150μm (A); 100μm **(B** **,C,** **F,** **G)**; 50μm in **D**,**E**.

**Figure 2:**
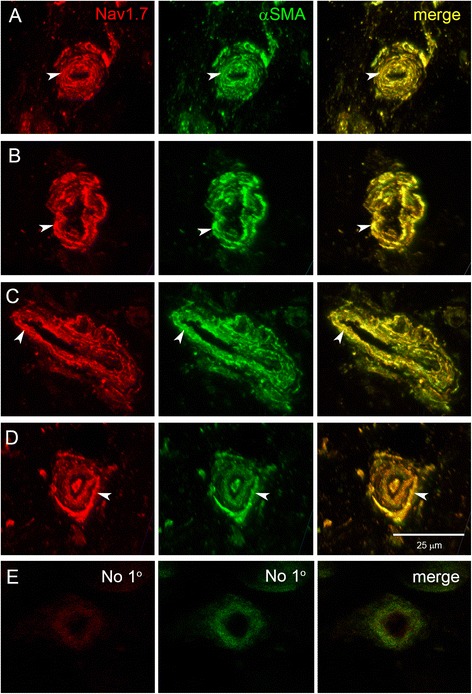
Nav1.7 expression in smooth muscle cells of deep dermis arterioles within skin from lateral malleolus of three healthy subjects. Smooth muscle cells (arrowheads) of the arteriole tunica media exhibit robust Nav1.7 (red) immunolabeling (antibody Nav1.7_Y_), which is co-localized with alpha smooth muscle actin (green). Skin samples from 3 healthy subjects (Subject 1: **A**; Subject 2: **B**,**C**; Subject 3: **D**) display similar patterns of Nav1.7 labeling in the smooth muscle cells of the dermal arterioles. Co-localization of Nav1.7 and alpha smooth muscle actin is yellow in the merged panels. **E**. Sections incubated without primary antibodies followed by secondary antibodies displayed background levels of immunofluorescence in skin vasculature.

**Figure 3:**
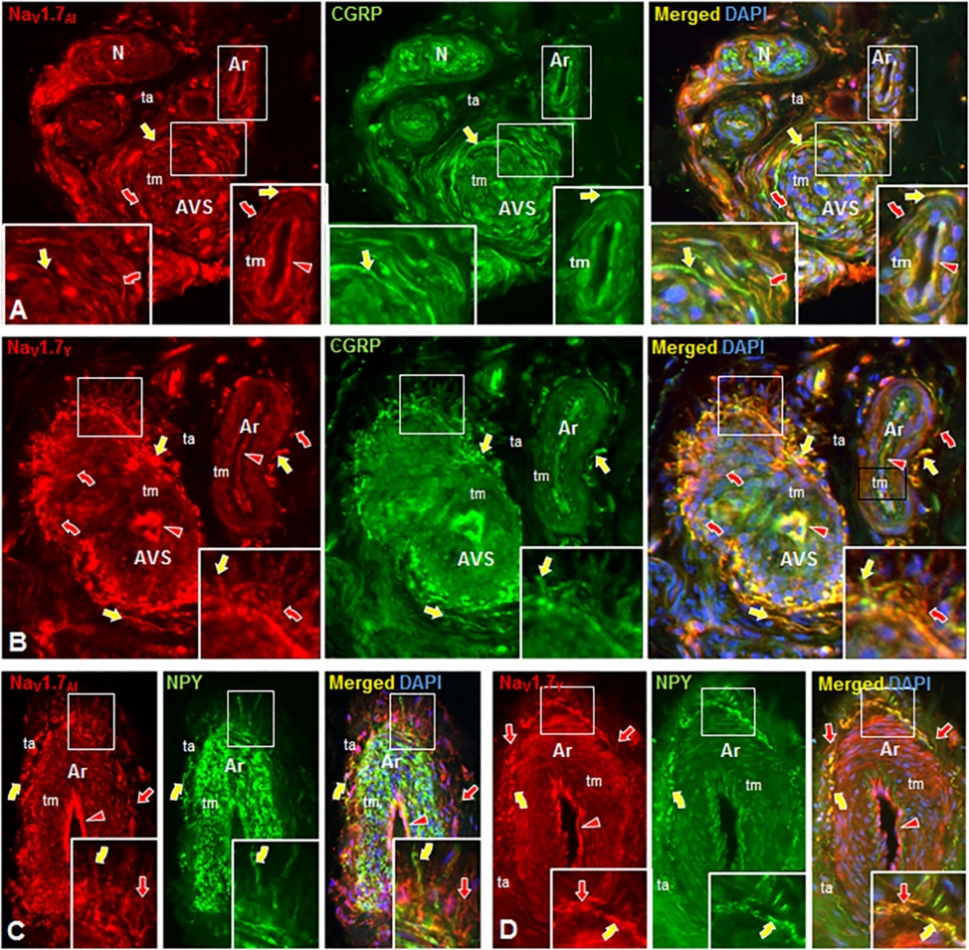
Digital fluorescence images of Nav1.7 immunolabeling (IL) of arterioles (Ar), arteriole-venule shunts (AVS) and associated innervation in normal human glabrous skin biopsies from the plantar foot **(A,C,D)** and palmar hand **(B)**. All sections are labeled with an Alomone **(A,C)** or Yale **(B,D)** rabbit anti-rat Nav1.7 antibody revealed by a donkey anti-rabbit Cy3-conjugated secondary antibody (red fluorescence). Secondary antibodies conjugated to Alexa 488 (green fluorescence) were used to assess co-labeling for peptidergic sensory innervation revealed with a sheep anti-human CGRP antibody **(A,B)** or noradrenergic sympathetic innervation revealed with a sheep anti-human NPY antibody **(C,D)**. Cell nuclei are labeled with DAPI (blue fluorescence). The left images in each panel show only the red fluorescence, the middle images only the green, and the right images the triple label combinations. Areas outlined in large white rectangles are 2X enlargement of the areas in the small rectangles. **A-D**. Nav1.7-IL is expressed on the endothelial cells of the tunica intima (red arrowheads) and on smooth muscle cells of the tunica media (tm). **A**,**B**. Peptidergic sensory innervation co-expresses Nav1.7-IL and CGRP-IL (yellow straight arrows). Other innervation labeled only with Nav1.7 (red curved arrows) is likely the noradrenergic sympathetic innervation that expresses NPY-IL as shown in **C** and **D**. **C**,**D**. Noradrenergic sympathetic innervation co-expresses Nav1.7-IL and NPY-IL (yellow curved arrows). Other innervation labeled with only Nav1.7 (red straight arrows) is likely the peptidergic sensory innervation that expresses CGRP-IL as shown in **A** and **B**. Scale Bar = 150 μm in **A** and **B**, 100 μm in **C** and **D**.

**Figure 4:**
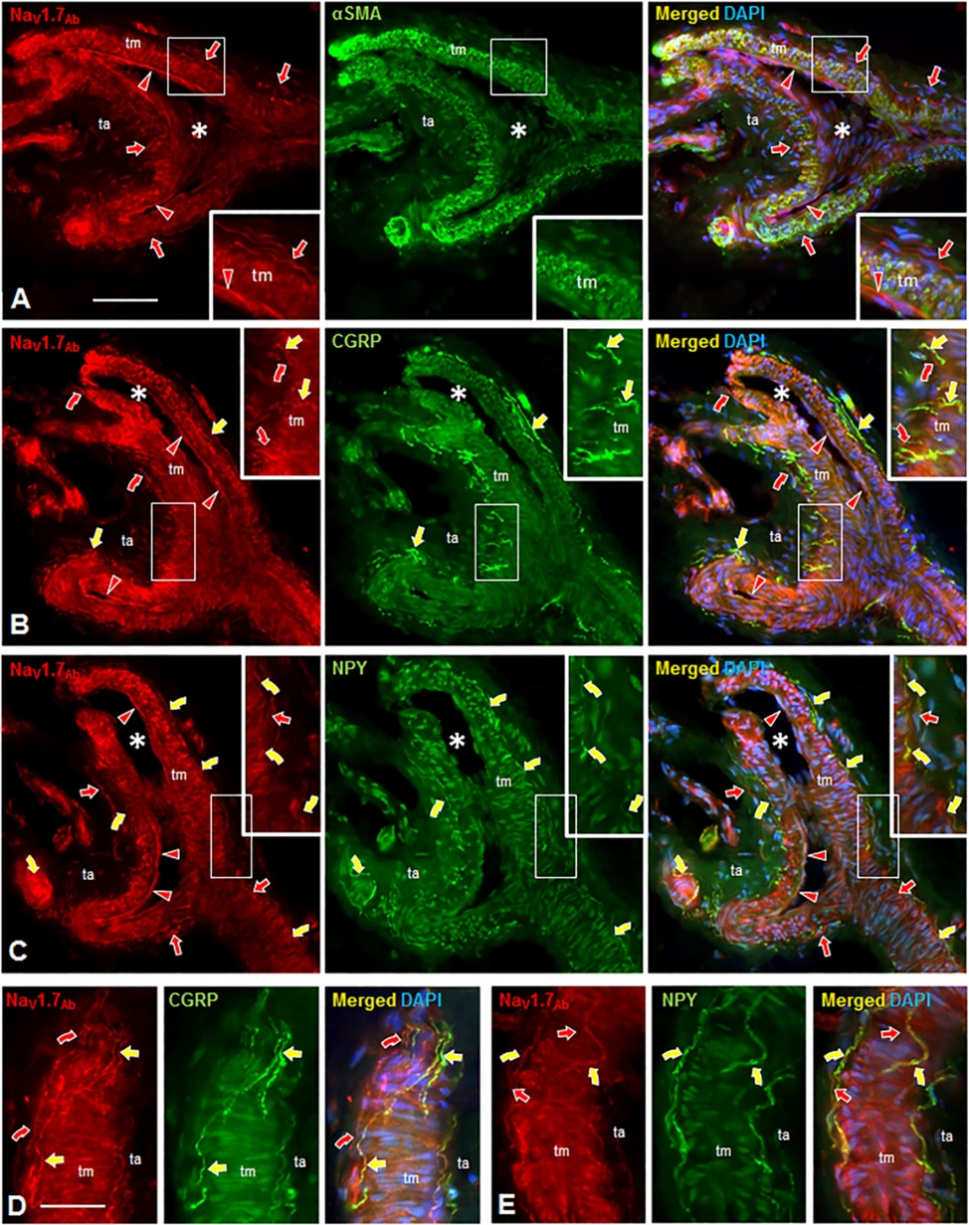
Nav1.7 immunolabeling (IL) of arterioles and associated innervation in normal human palmar glabrous skin biopsies, in alternating sections cut parallel to and through lumen (*) of branched arteriole **(A-C)** and parallel to arteriole, skimming the interface between tunica media (tm) and tunica adventitia (ta) **(D,E)**. All sections are labeled with Abcam anti-human Nav1.7 antibody (red). Secondary antibodies conjugated to Alexa 488 (green) were used to assess co-labeling for: smooth muscle cells revealed with mouse anti-α−smooth muscle actin antibody (αSMA, **A**); peptidergic sensory innervation revealed with sheep anti-CGRP antibody (yellow straight arrows, **B**,**D**); and noradrenergic sympathetic innervation revealed with sheep anti-NPY antibody (yellow curved arrows, **C**,**E**): Nuclei are labeled with DAPI (blue). Left images in each panel show only the red fluorescence, middle images only green, and right images the triple-label combinations. Areas outlined in large white rectangles **(A-C)** are 2X enlargements of areas in small rectangles. **A-E**.** A**. Nav1.7-IL is expressed on endothelial cells of tunica intima (red arrowheads) and smooth muscle cells of tm as confirmed by double-labeling with anti-αSMA. Nav1.7-IL is expressed on innervation (arrows) in ta, near and at the border with tm. **B**, **D**. Peptidergic sensory innervation co-expresses Nav1.7-IL and CGRP-IL (yellow straight arrows). Other innervation labeled only with Nav1.7 (red curved arrows) is likely noradrenergic sympathetic innervation that expresses NPY-IL **(C,E)**. **C**,**E**. Noradrenergic sympathetic innervation co-expresses Nav1.7-IL and NPY-IL (yellow curved arrows). Other innervation labeled with only Nav1.7 (red straight arrows) is likely peptidergic sensory innervation that expresses CGRP-IL as shown in **B** and **D**. Scale bar = 100 μm in **A-C**, 50 μm in **D** and **E**.

The arterioles and AVS were assessed in 14 μm sections of 3 mm glabrous skin punch biopsies taken from the hypothenar compartment of the hand and lateral margin of the foot from three normal male and eight normal human female subjects ranging in age from 21–74 years old. Three distinct polyclonal antibodies were used in these studies that were raised against two different sequences of rat Nav1.7 (Nav1.7_Al_ and Nav1.7_Y_) and one far removed sequence of human Nav1.7 (Nav1.7_Ab_), which yielded similar labeling of the extensive sensory and sympathetic innervation, smooth muscle cells of the tunica media, and endothelial cells of the tunica intima of resistance arterioles and arteriole-venule shunts (AVS) located in the deep dermis of glabrous skin (Figures 1, 2, 3 and 4). The innervation of arterioles and AVS was predominantly concentrated in the tunica adventitia in close proximity to the tunica media [27]. Consistent with previous descriptions [2,29], innervation is more extensive surrounding the AVS, which have an especially thick tunica media, than that surrounding arterioles (Figure 1); these features distinguish between these two vascular structures. Also, the lumina of AVS are typically occluded due to constriction of the thick muscular wall during fixation.

In all samples examined, Nav1.7 immunolabeling was intensely expressed throughout the tunica media of arterioles and AVS. Double-labeling with antibodies directed to Nav1.7 and αSMA, a marker of smooth muscle cells, confirmed the expression of Nav1.7 in tunica media smooth muscle cells within glabrous skin (Nav1.7_Al_: Figure 1B; Nav1.7_Y_: Figures 1C; Nav1.7_Ab_: Figure 4A). Double-labeling with Nav1.7_Y_ and αSMA was also examined in resistance arterioles in skin from the lateral malleolus of three additional healthy human subjects (Figure 2A-D). As exemplified in Figure 2A-D, there was extensive co-localization of Nav1.7 and αSMA in arteriole smooth muscle cells of skin biopsies from the lateral malleolus from each of these three different subjects.

#### Endothelial cells

Each of the three Nav1.7 antibodies (Nav1.7_Y_, Nav1.7_Al_ and Nav1.7_Ab_) used in our studies also labeled the endothelial cells that form the tunica intima lining of the arteriole and AVS lumina, which was confirmed by double-labeling for platelet endothelial cell adhesion molecule 1 (PECAM), a marker of endothelial cells (Figures 1D, E).

### Nav1.7 expression in fibers innervating cutaneous vascular structures

The neuronal marker, PGP 9.5, was utilized to identify innervation of resistance arterioles and AVS in the deep dermis in glabrous skin [2,27]. As shown in Figure 1A, double-labeling studies with antibodies to Nav1.7 and PGP 9.5 demonstrated that virtually all fibers innervating arteriole and AVS exhibited Nav1.7 immunolabeling (Figure 1A). It is likely that the PGP 9.5- and Nav1.7-positive fibers included both the sensory and sympathetic innervation. To distinguish sensory from sympathetic fibers that expressed Nav1.7, we performed double-immunolabeling studies with antibodies to calcitonin gene-related peptide (CGRP), which labels virtually all presumptive sensory innervation, and neuropeptide Y (NPY), which has previously been shown to label nearly all the noradrenergic sympathetic fibers innervating deep dermis arterioles [2,27].

#### CGRP-labeled fibers

Co-localization studies with all three Nav1.7 antibodies and a CGRP antibody demonstrated a subset of the Nav1.7-positive fibers that exhibited CGRP labeling in both arterioles and AVS (straight yellow arrows in Figures 3A, B, and 4B, D). In these double-labeling combinations, there were consistently some Nav1.7-positive fibers that did not display CGRP labeling (curved red arrows in Figures 3A,B and 4B,D), which were presumably sympathetic fibers.

#### NPY-labeled fibers

To identify the Nav1.7-positive sympathetic innervation of arterioles and AVS, tissue was reacted with antibodies to Nav1.7 and NPY. As shown in Figures 3 and 4, only a subset of fibers labeled with each of the three Nav1.7 antibodies exhibited NPY immunolabeling (yellow curved arrows in Figures 3C,D and 4C,E), indicating their identity as sympathetic fibers. As anticipated, there was consistently a subset of fibers that labeled with each of the three Nav1.7 antibodies that displayed an absence of NPY immunofluorescence, consistent with our demonstration of sensory fibers innervating the vasculature. In separate experiments, tissue was reacted with the anti-Nav1.7 antibody and a combination of CGRP and NPY antibodies (both raised in sheep). Virtually all Nav1.7-positive fibers were co-labeled with the combined CGRP and NPY antibodies (data not shown).

### Nav1.7 expression in the epidermis and upper dermis

**Figure 5:**
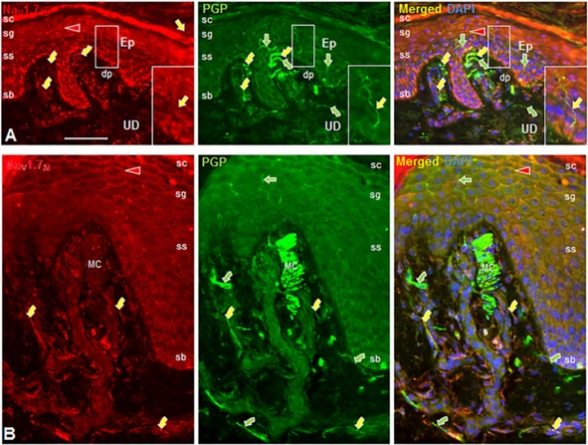
Digital fluorescence images of Nav1.7 (red) and PGP 9.5 (green) immunolabeling (IL) in the epidermis (Ep) and upper dermis (UD) biopsies of normal human palmar glabrous skin (**A**, Abcam anti-Nav1.7) and normal human plantar glabrous skin (**B**, Alomone anti-Nav1.7). Stratum corneum, sc; stratum granulosum, sg; stratum spinosum (ss); stratum basalis (sb), dermal papilla (dp). Straight arrows indicate epidermal sensory endings, curved arrows indicate small nerves and individual axons or endings in the upper dermis. The areas enclosed in the large rectangles are 2X enlargements of those in the smaller rectangles. Of all the innervation revealed by anti-PGP 9.5, only some express Nav1.7-IL (yellow straight and curved arrows) whereas other only express PGP 9.5-IL (green straight and curved arrows). Aβ-fiber innervation of a Meissner corpuscle (MC) has little if any Nav1.7-IL. Kertinocytes especially in stratum granulosum label for Nav1.7 (arrowheads) which has a more membranous distribution with the Alomone anti-Nav1.7 antibody, but more diffuse labeling with the Abcam anti-Nav1.7 antibody. Scale bar = 100 μm.

Given the high level of Nav1.7 labeling of the vascular smooth muscle cells, endothelial cells, and most of the vascular innervation in the upper dermis, we assessed the Nav1.7 and PGP 9.5 expression in the epidermis and upper dermis within the same sections (Figure 5). In this location, Nav1.7 immunolabeling was only detected on some of the innervation to the epidermis and upper dermis. Most of the innervation including the Aβ-fiber innervation of Meissner corpuscles lacked Nav1.7 labeling. Nav1.7 expression was detected among keratinocytes predominantly in the stratum granulosum as we had reported previously [30].

## Discussion

In this study, we demonstrate that sodium channel Nav1.7 is present within vascular myocytes and endothelium of arterioles and arteriole-venule shunts (AVS) of human skin, and in virtually all of the innervation to these resistance vessels, which consists of a dense convergence of sympathetic and sensory innervation [2,27,31]. Our demonstration of Nav1.7 co-expression with α-smooth muscle actin throughout the tunica media of arterioles and AVS within human skin is the first *in situ* documentation of the expression of Nav1.7 by smooth muscle cells and is consistent with prior reports of Na_V_1.7 expression on cultured myocytes dissociated from human aorta, pulmonary and brochiole arteries and murine aorta [32-35]. Our observation of Nav1.7-immunloabeling on the endothelial cells of the tunica intima is also consistent with prior RT-PCR detection of Nav1.7 in cultured vascular endothelial cells harvested from human umbilical cord veins, where a role in Nav1.7 regulation of angiogenesis has been suggested [36].

Consistent with previous reports of Nav1.7 on neurons in sympathetic ganglia of rats, Nav1.7 co-localized with NPY which is known to be co-expressed and released from noradrenergic (NA) sympathetic innervation, especially under sustained activation to supplement the vasoconstrictive properties of noradrenalin. Therefore, our results indicate that Nav1.7 is indeed present within the NA sympathetic [37-39] innervation of human cutaneous arterioles and AVS and presumably plays a role in regulating their sympathetically- mediated constriction.

We also observed that Na_V_1.7 is co-expressed separately with CGRP on virtually all the remaining innervation of the cutaneous arterioles and AVS, which is likely supplied by sensory neurons in the dorsal root ganglia (DRG) [37,38,40,41]. This presumed sensory innervation consists of several immunocytochemically distinct subtypes of C- and Aδ-fibers that co-express Substance P [31,42,43]. These results are consistent with prior observations that Nav1.7 is expressed on many small-to-medium size neurons in rat DRG, of which most co-express CGRP and Substance P [42,43]. Thus, at least some of these Nav1.7-positive peptidergic DRG neurons are the likely source of virtually all the innervation to cutaneous resistance vessels. Although CGRP and SP have been implicated in inflammatory pain, little is known about the specific sensory functions of the C- versus Aδ-fiber innervation of cutaneous resistance vessels of which further subtypes of these fibers have been identified by differential expression of other molecular characteristics such as TrpV1, ASIC3 and H3R [1,44,45]. Vascular terminals of these sensory fibers have also been shown to release CGRP and SP which are potent vasodilators [37,38,40,46-49]. Thus, Nav1.7 expression in these different varieties of sensory vascular fibers could presumably impacts their sensory as well as vasodilatory functions.

While Nav1.7 mutations associated with IEM are known to be gain-of-function at the channel level, enhancing activation in the case of IEM [14,50] or impairing fast-inactivation in PEPD [16,17], these mutations have divergent effects on different types of neurons that express the Nav1.7 channel. It is well-established that Nav1.7 mutations associated with IEM [50,51] produce hyperexcitability in DRG neurons. In contrast, these mutations produce hypoexcitability in sympathetic ganglion neurons [25,26]. The opposing effects of these Nav1.7 mutations reflect the presence in DRG neurons, but not in sympathetic ganglion neurons, of the Nav1.8 channel, which is relatively resistant to depolarization and supports repetitive firing in response to sustained depolarization [52]. As a result of hyperpolarized activation, these Nav1.7 mutations produce an enhanced window current, which depolarizes neurons [51]. This depolarization brings membrane potential closer to the threshold for activation for Nav1.8, and therefore is pro-excitatory within DRG neurons; in contrast, the depolarization inactivates all of the sodium channels within sympathetic ganglion neurons, which do not contain Nav1.8, thereby reducing excitability in these cells [25].

The opposing effects of Nav1.7 signaling on sensory and sympathetic neurons align well with, and may contribute to, antagonistic vasodilatation and vasoconstriction roles, respectively, of these two types of innervation [2,27]. The vasoconstrictive function of noradrenergic sympathetic innervation on resistance vessels is well-established [37-39]. The mechanisms of vasodilatation have been more problematic (see [2]). Cholinergic innervation from discrete parasympathetic ganglia is a primary source of neurogenic vasodilatation of arterioles in the brain and face [4,5,53,54]. However, such cholinergic innervation is minimal to the cutaneous arterioles and AVS in the hands, feet, and other areas where vasodilatation has widely been regarded as a passive relaxation consequent to reduced NA sympathetic activity [4,5,27]. The potential role of vascular sensory fibers in neurogenic activation of cutaneous vasodilatation has received little attention despite the fact that the vascular terminals release CGRP and SP which are potent vasodilators [55,56], possibly acting through antidromic activity among vascular afferents [46,48,49,57]. Moreover, activation of TrpV1 and ASIC3, which are co-expressed on many peptidergic sensory neurons, has been shown to promote CGRP release [44,58], whereas activation of co-expressed H3R has been implicated in inhibiting CGRP release [43]. These observations suggest the presence of local tissue regulators of sensory-mediated vasodilatation. Albrecht et al. [2] observed that nearly all of the sensory innervation to arterioles and AVS in the glabrous palmar skin of humans also co-express the α2C adrenergic receptor which has been shown to inhibit CGRP release [59-63]. This suggests that NA sympathetic activation of vasoconstriction may also involve inhibition of sensory- mediated vasodilatation. On the basis of our results, we hypothesize that Nav1.7 may be involved in multiple vasodilatory mechanisms, both enhancing the activity of vasodilatory sensory innervation and concomitantly reducing the activity of vasoconstrictive noradrenergic innervation.

Our detection of Nav1.7 in the myocytes of the tunica media and endothelial cells of the tunica intima in the cutaneous arterioles and AVS raises the possibility of some other intriguing contributions to vasoregulation. Vascular myocytes and endothelial cells are not known to express Nav1.8. Therefore, we propose that Nav1.7 mutations that depolarize resting potential may produce hypoexcitability of intracutaneous vascular myocytes which would favor vasodilatation. On the other hand, endothelial cells release nitric oxide which contributes to vasodilation [64-66], so a reduction in their activation might be expected to favor vasoconstriction.

Little is known about the specific sensory roles of the various molecularly distinct subtypes types of C- and Aδ- sensory fibers that terminate on the cutaneous arterioles and AVS of which nearly all express CGRP, SP and Nav1.7. Some are implicated as nociceptors contributing to inflammatory pain through the release of CGRP and SP terminals in the CNS involving presumptive pain pathways. Others are implicated as metaboreceptors expressing ASIC3 which is preferentially activated by lactic acid [58]. Recently Bowsher et al. [27] showed evidence that the different subsets of vascular sensory innervation may contribute to a functionally adequate conscious capacity to spatially distinguish between a variety of non-noxious and noxious tactile stimuli. On the opposite extreme, Albrecht et al. [2] found that nearly all of a sizeable cohort of female fibromyalgia patients had an extremely significant excessive innervation, especially the sensory fibers selectively associated with the AVS in the palms of their hands. This neurovascular pathology is logically consistent with extreme palmar tenderness, especially bothersome at colder temperatures, and widespread deep pain possibly due to systemic consequences of vasodysregulation. A disproportionate over-representation of sensory innervation expressing α2C receptors could provide a rationale for use of SNRIs which could enhance an inhibitory regulation by the convergent NA sympathetic innervation.

PEPD, which is associated with Nav1.7 mutations that impair fast-inactivation, produces attacks characterized by a pattern of pain (perineal in young patients, becoming periocular or perimandibular in adults) and of skin reddening (often of the face or torso, sometimes in a harlequin pattern that affects only one-half of the body) [17,19] that differs from the pattern in IEM (pain and reddening of the distal limbs) [15]. Current-clamp studies that have been carried out on PEPD mutations thus far have demonstrated hyperexcitability of DRG neurons expressing the mutant channels, but have not demonstrated depolarization of resting potential as a result of expression of these mutations [67,68]. Since these mutations do not depolarize resting potential, they would not be expected to reduce neuronal excitability in cells lacking Nav1.8 such as sympathetic ganglion neurons [25]. As a result of the impairment that they confer on inactivation, these PEPD mutations are predicted to produce hyperexcitability within sympathetic ganglion neurons and their axons. A differential effect of IEM and PEPD mutations on sympathetic ganglion neurons might contribute to the different pattern of vasomotor symptomology in patients with PEPD, compared to those with inherited erythromelalgia [15,17,19].

Taken together, our results demonstrate the presence of Nav1.7 in vascular myocytes, endothelium, and sensory and sympathetic axons innervating the vasculature in human skin, and support the idea that vasogenic and neurogenic mechanisms both contribute to skin reddening in disorders due to Nav1.7 mutations such as IEM and PEPD.

## Methods

### Human tissue

The analyses documented in Figures 1, 3, 4, and 5 were conducted on 3 mm punch biopsies obtained from 3 normal male and 8 normal female subjects ranging in age from 21–74 years old in obtained in accordance with IRB approval at St. Peters Hospital in Albany, New York. The biopsies were fixed by immersion for 4 hours in ice-cold 4% paraformaldehyde in 0.1 M phosphate buffered saline (PBS), pH 7.4, and were cryoprotected and frozen sectioned at 14 μm thickness by cryostat in a plane perpendicular to the epidermal surface. Alternating sections were thaw-mounted in serial order rotating across as many as 20 slides such that each slide had as many as 15 sections from equally spaced intervals through the biopsy. The morphological and immunocytochemical characteristics of the innervation had previously been assessed and published on some of the slides from each biopsy using a variety of antibodies targeting antigens implicated in various neural properties [27,69]. Slides from some of the biopsies had previously been processed with a commercial rabbit polyclonal antibody directed against rat Nav1.7 (see below, Alomone Labs, ASC-008) for a study [30] of the epidermis and upper dermis which did not examine the vascular innervation in the same sections. All of the processed and unprocessed slides had been stored at −20°C under glycerin mounted coverslips which we have floated off in PBS for new or additional double immunolabeling after at least 10 years in archive. The advantage of this archiving system is that such alternating slides can be used for follow up analyses on biopsies that have been previously well characterized for other purposes.

For this study, we assessed sections from palmar and plantar glabrous skin which have a high density and variety of innervation types and a high probability (75%) of containing densely innervated arterioles and AVS which is the focus of this study.

Additional skin tissue that was imaged for Figure 2 was obtained by 3 mm punch biopsies 10 cm above the lateral malleolus from 3 healthy volunteers (age: 30, 32 and 50 years) in accordance with IRB approval at Maastricht University and VA Connecticut Healthcare System, West Haven. The samples were fixed for 30 minutes in 4% paraformaldehyde in 0.14 M Sorensen’s phosphate buffer, rinsed with PBS, cryoprotected with 30% sucrose in PBS, and rapidly frozen. Twelve μm cryosections were collected on SuperFrost Plus glass slides (Fischer) and processed for immunocytochemical analysis.

### Immunocytochemistry

Three affinity purified antibodies generated to different amino acid sequences in rat and human Nav1.7 were utilized in these studies: rabbit polyclonal Nav1.7_Al_: Alomone Labs, ASC-008, rat 446–460 aa sequence, 1:100; rabbit polyclonal Nav1.7_Y_: Y083, [70], rat 514–532 aa sequence, 1:250; and rabbit polyclonal Nav1.7_Ab_: Abcam Inc, ab85167, human 1000–1100 aa sequence, 1:500. All three Nav1.7 antibodies exhibited similar patterns of labeling. To identify structures labeled with the Nav1.7 antibodies, tissue was double-labeled with Nav1.7 antibody and antibodies against human protein-gene-product 9.5 (PGP 9.5, rabbit polyclonal, UltraClone LTD, RA95101, 1:800), which labels all known types of nerve fibers; human calcitonin gene-related peptide (CGRP, sheep polyclonal, Abcam Inc, Ab22560, 1:500), which labels nearly all types of arterial C- and Aδ-fiber innervation; human neuropeptide Y (NPY, sheep polyclonal, EMD Millipore Corp, AB1583, 1:800), which labels noradrenergic sympathetic innervation; alpha smooth muscle actin (αSMA, mouse monoclonal, Abcam Inc, Ab7817, 1:100), and human platelet/endothelial cell adhesion molecule (PECAM, mouse monoclonal, DAKO, M0823, 1:50). Secondary antibodies consisted of donkey anti-rabbit IgG-Cy3 (Jackson ImmunoResearch, 711-165-152, 1:500) and donkey anti-mouse and –sheep IgG conjugated to Alexa 488 (Invitrogen, A21202 and A11015, 1:250) and were processed as previously described [2,70]. Some sections were counterstained with DAPI to reveal cell nuclei.

Control experiments included omission of primary antibodies, which displayed only background levels of immunofluorescence among all components of the skin including not only the vessels and innervation in the deep dermis (Figures 1F and 2E) but also in the epidermis and upper dermis (Figure 1G). The Alomone Nav1.7 immunolabeling pattern had been previously validated by preabsorption of the antibody with the cognate peptide and by *in situ* hybridization labeling pattern [30].

### Image acquisition

Epifluorescent images for Figures 1, 3, 4, and 5 were captured utilizing an Olympus BX51-WI microscope equipped with conventional fluorescence filters (Cy3: 528–553 nm excitation, 590–650 nm emission; Cy2/Alexa 488: 460–500 nm excitation, 510–560 nm emission), a Hamamatsu ER, DVC high-speed camera, linear focus encoder, and a 3-axis motorized stage system interfaced with Neurolucida software (MBF Bioscience, Essex, VT). Images were composed with Photoshop (Adobe, San Jose, CA) with minimal contrast enhancement from the original images.

Images of fluorescent-labeled tissue for Figure 2 were accrued as z-stacks with a Nikon C1*si* confocal microscope (Nikon USA, Melville, NY) operating with frame lambda (sequential) mode and saturation indicator to prevent possible bleed-through between channels. Z-stack images were appropriately rotated to visualize arteriole lumena and were composed and processed with Photoshop.
